# Development of hypoallergenic variants of the major horse allergen Equ c 1 for immunotherapy by rational structure based engineering

**DOI:** 10.1038/s41598-019-56812-1

**Published:** 2019-12-27

**Authors:** Jaana Haka, Merja H. Niemi, Pekka Mattila, Janne Jänis, Kristiina Takkinen, Juha Rouvinen

**Affiliations:** 1 Desentum Ltd, Kivipylväänkuja 5, 02940 Espoo, Finland; 2 Department of Chemistry, University of Eastern Finland, PO Box 111, 80101 Joensuu, Finland; 3 VTT Technical Research Centre of Finland, PO Box 1000, 02044 Espoo, Finland

**Keywords:** Protein vaccines, Applied immunology, Vaccines, Protein design, Immunochemistry, Antigen processing and presentation, Signal transduction

## Abstract

The use of recombinant allergens is a promising approach in allergen-specific immunotherapy (AIT). Considerable limitation, however, has been the ability of recombinant allergens to activate effector cells leading to allergic reactions. Recombinant hypoallergens with preserved protein folding and capacity to induce protective IgG antibodies binding effectively to the native allergen upon sensitization would be beneficial for safer AIT. In this study, hypoallergen variants of the major horse allergen Equ c 1 were designed by introducing one point mutation on the putative IgE epitope region and two mutations on the monomer-monomer interface of Equ c 1 dimer. The recombinant Equ c 1 wild type and the variants were produced and purified to homogeneity, characterized by size-exclusion ultra-high performance liquid chromatography and ultra-high resolution mass spectrometry. The IgE-binding profiles were analyzed by a competitive immunoassay and the biological activity by a histamine release assay using sera from horse allergic individuals. Two Equ c 1 variants, Triple 2 (V47K + V110E + F112K) and Triple 3 (E21Y + V110E + F112K) showed lower allergen-specific IgE-binding capacity and decreased capability to release histamine from basophils *in vitro* when using sera from six allergic individuals. Triple 3 showed higher reduction than Triple 2 in IgE-binding (5.5 fold) and in histamine release (15.7 fold) compared to wild type Equ c 1. Mutations designed on the putative IgE epitope region and monomer-monomer interface of Equ c 1 resulted in decreased dimerization, a lower IgE-binding capacity and a reduced triggering of an allergic response *in vitro*.

## Introduction

Currently, allergen-specific immunotherapy (AIT) is based on allergen extracts obtained from natural sources. It has been argued over a decade that these extracts have shortages mainly because of their inconsistent characteristics and quality. Due to the inherent heterogeneous property of natural allergen sources and extract producing methods, the extracts contain individual allergens with variable content and concentration but also with undefined biological activity. In addition, extracts contain large variety of non-allergenic materials and contaminants which nature is usually unknown and which may have, for example, toxic or undesired immunomodulatory effects. Recombinant allergens produced under good manufacturing practices (GMP) and adequately characterized to show high purity and consistent quality offer several advantages over extracts as medicinal products, most importantly due to the accurate dose administration during AIT^1^.

AIT causes various immunological changes especially in T cells leading to an early increase in allergen-specific IgE response, which decline later and an increase in the allergen-specific protective IgG response, especially subtype IgG_4_. Protective IgG antibodies capture invading allergen molecules before they reach the effector-cell bound IgE antibodies thus preventing activation of mast cells or basophils^2^.

A number of clinical trials with recombinant allergens, their variants or allergen-derived peptides with or without carrier proteins have been conducted^1^. Peptides or their derivatives have been favored due to their minimal ability to activate effector cells that offers substantial safety factor. However, peptides are prone to rapid degradation by tissue and serum proteases and the use of peptide antigens in immunization experiments is consistently less effective compared to full length proteins^3^. The use of peptides in immunization generates antibodies which usually recognize denatured (unfolded) proteins whereas immunization with correctly folded proteins produce antibodies which bind to natively folded biologically active proteins^3^. One approach is to use a peptide carrier strategy which has been reported to induce allergen-specific antibodies that recognize also the peptides in the context of the complete allergens^4^.

IgE epitopes are conformational consisting usually several non-continuous stretches (segments) of the polypeptide chain forming a specific three-dimensional structure on the surface of the allergen. The most accurate method to determine an IgE epitope is to co-crystallize the allergen in a complex with the Fab-fragment of patient-derived IgE-antibody^5^. Due to rather challenging techniques associated with isolation of allergen specific IgE antibodies and co-crystallization of protein complexes, only two such IgE/Fab-allergen complex structures have been determined so far; for bovine milk allergen Bos d 5 and for timothy grass pollen allergen Phl p 2. Interestingly, in both of the studies the overall spatial shape of the IgE epitope was flat consisting mainly of secondary structure elements (β-strands) differing from IgG epitopes which are typically located in the protruding regions of allergens^6,7^.

The immunocomplex structure of Bos d 5 revealed also that the allergen formed a dimer to which two identical Fab fragments were simultaneously bound indicating that two monoclonal IgE antibodies would be able to form a productive complex with a dimeric allergen to active mast cell or basophil^6^. In our subsequent studies involving a number of allergens with their crystal structures and mass spectrometric analyses, we have discovered that majority of the allergens are able to form weak or transient dimers depending on the allergen concentration^8^. We have proposed a sequential model for mast cell or basophil activation in which monomeric allergen binds first to the FcεRI bound IgE on the cell surface and subsequent dimerization is strongly boosted due to dimensional reduction (delocalization) on the cell surface leading to a signal transduction^9^.

The strategy to design hypoallergenic variants which have a diminished capability to form allergen-IgE complexes on the surface of an effector cell would thus include mutations on the flat IgE epitope and/or monomer-monomer interface. We have implemented these ideas in designing of two hypoallergenic variants of the major horse allergen Equ c 1. Horse allergy occurs in people who regularly work with horses, either professionally or for recreational purposes, and also in people indirectly exposed to horses. The prevalence of horse sensitization in occupational settings varies between 3.6 and 16.5% and people with a respiratory allergy have about 5% sensitization rate^10^. Horse allergy is induced by exposure to the horse allergens, such as Equ c 1, c2, c3, c4 and c 5. Of these Equ c 1, the major horse allergen, is a hair dander protein and the most important from a clinical perspective. Equ c 1 is responsible for about 80% of anti-horse IgE antibody response in patients who have been exposed to horse allergens^11^. Immonen *et al*. have also analyzed T-cell epitopes of Equ c 1 and found two major, partially overlapping epitopes covering the amino acid residues 140–163 (Supplementary Fig. S1)^12^.

Equ c 1 belongs to the lipocalin protein family, which members typically transport small hydrophobic ligands^13^. The lipocalin family contains a number of animal dander allergens but also previously mentioned bovine milk allergen Bos d 5. Equ c 1 allergen consists of 187 amino acid residues and has one disulphide bridge. The crystal structure has revealed a dimeric structure with a large monomer-monomer interface (1025 Å^2^) suggesting that dimeric form is predominant^14^.

In this study, hypoallergenic variants of Equ c 1 were designed by targeting mutations to the (I) putative IgE epitope region aiming to reduce the binding of IgE antibodies and to the (II) monomer-monomer interface aiming to reduce dimerization of Equ c 1 monomers bound to the IgE-FcɛRI receptor complex yet aiming to preserve the three-dimensional fold highly resembling that of the wild type allergen. Two variants (Triple 2 and 3) of Equ c 1 having one IgE-epitope mutation and two mutations on the monomer-monomer interface allergen were designed. The recombinant Equ c 1 wild type (wt) and variants were produced in *E. coli*, purified to homogeneity and validated by size-exclusion ultra-high-performance liquid chromatography (SE-UHPLC) and ultra-high resolution (FTICR) mass spectrometry (MS). IgE-binding profiles of allergens were analyzed by a competitive immunoassay and biological activity by a histamine release assay (HRA).

## Results

### Design of hypoallergenic variants

**Figure 1:**
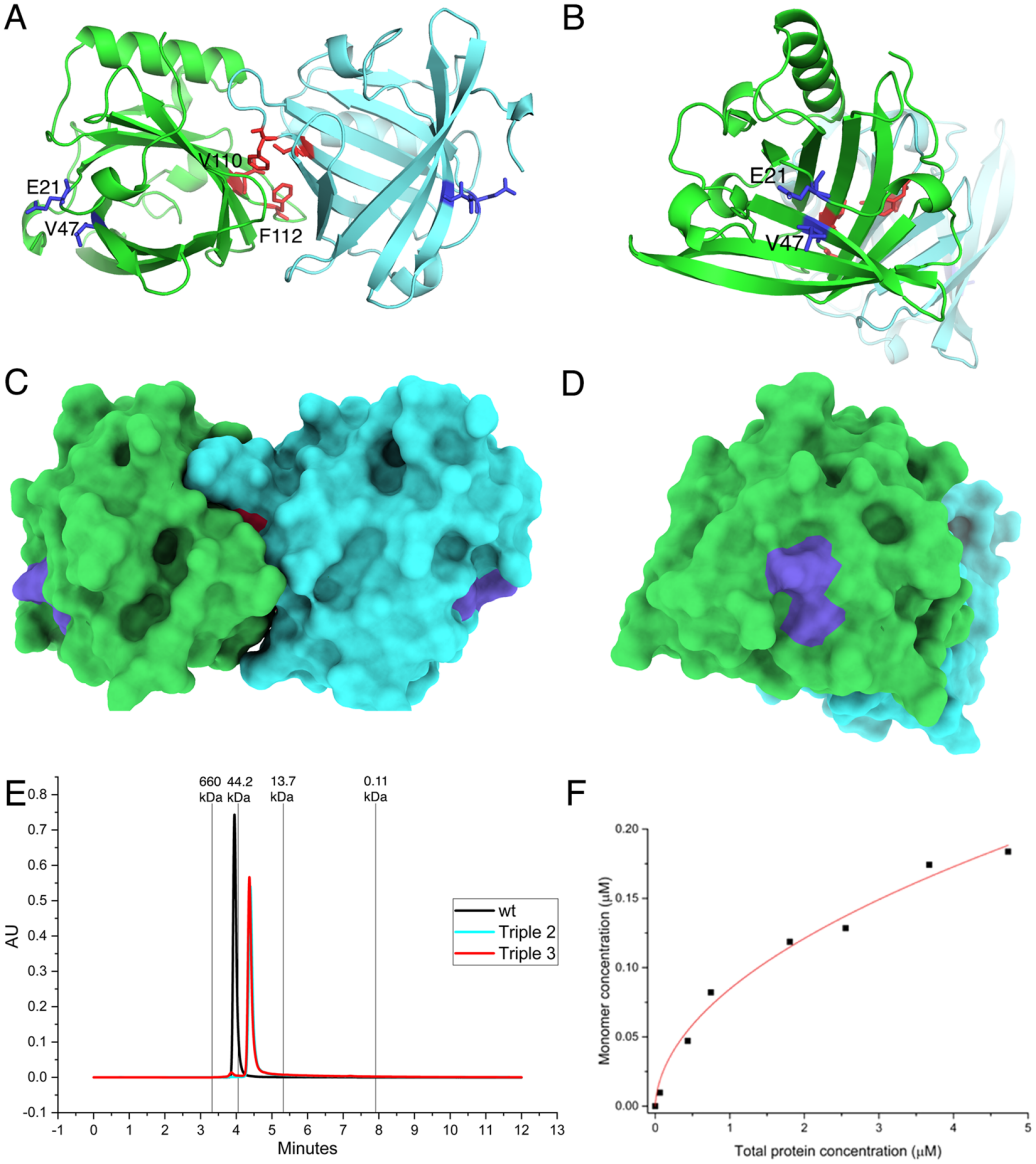
Molecular structure of Equ c 1 dimer. (**A**) Ribbon representation, monomer A (in green) and molecule B (in cyan). (**B**) as in A but rotated about 90° along y-axis. Residues which are mutated in monomer-monomer interface are shown as red sticks, epitope mutations as blue sticks. (**C,D**) The molecular surface of Equ c 1 dimer in two orientations. (**E**) The SE-UHPLC elution chromatograms of rEqu c 1 wt (in black), Triple 2 (in cyan, overlapped with Triple 3) and Triple 3 (in red) mutants as merged chromatograms. The elution retention times of the molecular weight standards are indicated in the figure. (**F**) rEqu c 1 wt monomer concentration as a function of total protein concentration as calculated from the native mass spectra.

Mutations were designed both on the monomer-monomer interface and the putative IgE epitope of Equ c 1. The three-dimensional coordinates of Equ c 1 wild type were downloaded from the protein data bank (code 1EW3). The crystal structure contained one molecule in an asymmetric unit. The other half of the Equ c 1 dimer was formed by using the crystallographic symmetry operator. The extensive monomer-monomer interface (area 1024 Å^2^) was studied by testing different mutations in the middle of the interface* in silico*. The effect of mutations were predicted by evaluating their ability to break molecular surface complementarity and hydrophobic interactions as well as hydrogen bonds and charged interactions between protein molecules. Hydrophobic groups do not interact well with polar groups, especially with charged ones. Therefore, substitution of hydrophobic residues with charged residues or vice versa should have more substantial effect to diminish protein-protein interaction. It was concluded that V110E and F112K mutations would be the most effective to disrupt the dimer formation of Equ c 1. The IgE epitopes of Equ c 1 are undefined, hence the putative IgE epitope was deduced by searching flat areas on the molecular surface of either side of the two-fold axis of symmetry of the Equ c 1 dimer by using PyMOL. Similarly, it was predicted that mutations E21Y and V47K would be the most promising candidates to achieve the decreased binding of IgE antibodies to Equ c 1. Two variants were selected for further studies: Triple 2 (V47K + V110E + F112K) and Triple 3 (E21Y + V110E + F112K) (Fig. 1A–D). Triple 1 variant with mutations G20V (putative IgE-epitope) combined to V110E and F112K was also designed, expressed in *E.coli* and purified with similar protocols as Triple 2 and 3. Expression level of Triple 1 was good, but it was much more prone to precipitate during adjustment of pH and concentration steps compared to Triple 2 and 3 and thus was not characterized further.

### Characterization of rEqu c 1 allergens by SE-UHPLC

SE-UHPLC results show that rEqu c 1 wt, Triple 2 and 3 variants eluted with retention times of 4.0, 4.4 and 4.4 minutes, respectively (Fig. 1E). Compared to protein standard elution chromatogram Triple variants eluted mainly as monomeric forms whereas wild type as dimeric form when injected at concentration of 40 µM.

### Mass spectrometry of rEqu c 1 wt and the hypoallergenic variants

**Figure 2:**
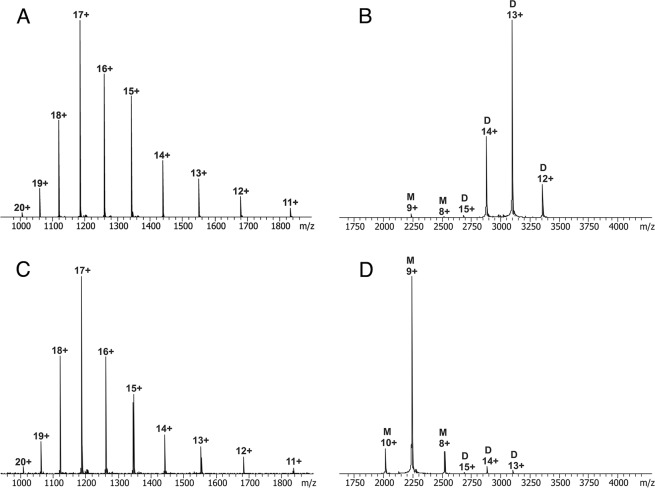
The high resolution mass spectra for rEqu c 1 wt in denatured (**A**) and native form (**B**), and for Triple 3 in denatured form (**C**) and in native form (**D**). Numbers refer to the charge states. Native spectra were measured at 40 μM protein concentrations and monomeric and dimeric peaks are labelled (M or D).

The high-resolution mass spectrometric characterization of wild-type allergen and the variants Triple 2 and Triple 3 in denaturing solution conditions was used to determine the accurate molecular masses and to observe possible modifications in the protein preparations (Figs. 2A,C and S2A, SDS-PAGE analysis of the purifed Equ c 1 allergens is also shown in Fig. S3). The experimentally determined molecular mass (most abundant isotopic mass, averaged over the charge state distribution) of cytoplasmic rEqu c 1 wt was 20,166.04 ± 0.01 Da, which corresponds well with the theoretical molecular mass (20,165.99 Da) of the allergen with a disulfide bridge between Cys68 and Cys161. Similarly, the molecular masses of cytoplasmic Triple 2 (20,206.03 ± 0.04 Da) and Triple 3 (20,211.02 ± 0.04 Da) corresponded precisely with the theoretical masses calculated from the amino acid sequences of the hypoallergenic variants (20,206.02 Da and 20,211.01 Da for Triple 2 and Triple 3, respectively). The r Equ c 1 wt and Triple allergens were detected to be highly pure and homogeneous.

Native mass spectrometry was used to study the oligomeric state of proteins and to investigate the correct folding of the hypoallergenic variants compared with rEqu c 1 wt. The native MS showed that at the protein concentration of 40 μM, the rEqu c 1 wt allergen predominantly exists as a dimeric form, whereas the rEqu c 1 variants are mainly monomeric. The native ESI FT-ICR mass spectra displayed a limited number of low charge states, indicating that both the wild-type allergen and the variants have similar, tightly folded structures (Fig. 2B,D).

To quantify dimerization of rEqu c 1 wt, monomer-dimer ratios were measured over a range of allergen concentrations (0.1–4.8 μM) by using ESI MS. The fitted curve of the free monomeric allergen concentration against the total allergen concentration is shown in Fig. 1F. The calculated dissociation constant *K*_*d*_ for rEqu c 1 wt dimer was 15.6 ± 0.8 nM which indicates strong tendency for Equ c 1 to dimerize but showing also a nature of this allergen to dissociate to monomers at nanomolar concentrations. It was not possible to perform similar measurements at different concentrations for Triple 2 and 3 because the signal for dimer was very low. However, by using intensities of monomer and dimer of Triple 3 at a single concentration (Fig. 2D), it can be estimated that *K*_*d*_ for Triple 3 dimer would be roughly 0.8 mM.

### IgE binding analysis

**Figure 3:**
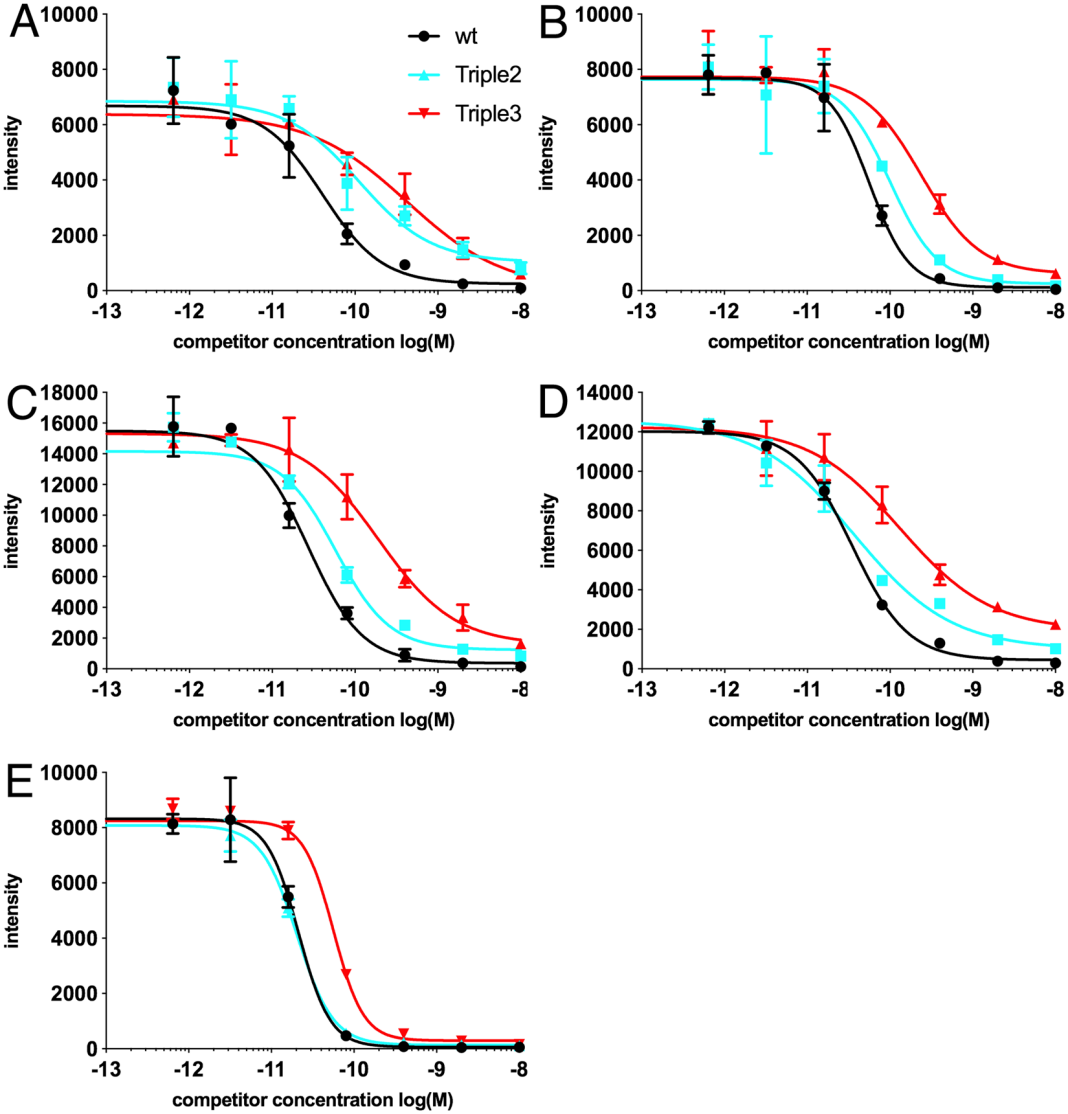
The competitive immunoassay measuring (fluorescence intensity, y-axis) the inhibition of serum IgE binding to rEqu c 1 wt obtained from five horse allergic patients (**A–E**) with increasing soluble concentrations (x-axis) of rEqu c 1 wt, Triple 2 and Triple 3.

The serum IgE binding to the recombinant Equ c 1 wild type and Triple variants was analyzed by a miniaturized competitive immunoassay (Fig. 3). The wild-type Equ c 1 was used to capture anti-Equ c 1 IgE antibodies from a human serum sample as increasing amounts of soluble allergen (wt, Triple 2 or Triple 3) was used to inhibit the binding. Soluble allergen competed the binding of IgE antibodies to the immobilized wild type Equ c 1. The binding of serum IgE was analyzed from five horse allergic individuals and one non-allergic individual. The obtained data allowed us to calculate the half maximal inhibitory concentrations (*IC*_50_) by fitting a curve to the dilution points of the dilution series of the competing allergen. The trend in the resulting curves were similar irrespective of the spotted concentration used. The results presented here are derived with the spotted wild type allergen concentration of 95 µg/ml.

The calculated *IC*_50_ values for rEqu c 1 wild type were similar for all sera with the median value of 35 pM (Table 1). The analyses showed reduced binding of allergen-specific IgEs for Triple variants 2 and 3 compared to rEqu c 1 wt (Fig. 3), medians 59 pM and 191 pM, respectively. The reduction was more significant for Triple 3 in all five serum samples. The results showed statistically significant differences between the two variants and wild type Equ c1 in three out of the eight comparisons (Table 1).

**Table 1:** The half maximal inhibitor concentrations (*IC*_50_) in a competitive immunoassay measuring the IgE binding and the half maximal effective concentrations (*EC*_50_) in a histamine release assay.

Serum	Equ c 1 ISU	*IC*_50_ [pM]			*P* value (vs wt)		*EC*_50_ [pM]			*P* value (vs wt)	
		rEqu c 1 wt	Triple 2	Triple 3	Triple 2	Triple 3	rEqu c 1 wt	Triple 2	Triple 3	Triple 2	Triple 3
A (F)	11	41	113	427	**0.009**	0.066	7	110	290	**0.004**	**0.005**
B (M)	14	56	100	240	0.181	0.059	5	240	420	**0.017**	**0.016**
C (F)	16	27	59	191	0.376	**0.041**	20	220	520	**0.030**	**0.033**
D (F)	22	35	37	133	0.074	**0.018**	40	180	260	0.280	0.244
E (M)	ND	21	20	56	0.346	0.142	42	840	3700	**0.022**	**0.018**
F	ND						45	720	1100	**0.029**	**0.032**
*median*		35	59	191			30	230	470		

Statistical analysis of differences are estimated by using *P* values. Significant differences (*P* < 0.05) are in bold. The quantity of rEqu c 1 specific IgE in serum, ISU-units (ISAC Standardized Unit), has been measured with the semiquantitative ImmunoCAP ISAC technology (Thermo Fisher). (F) female, (M) male.

### Histamine release of rEqu c 1 allergens

**Figure 4:**
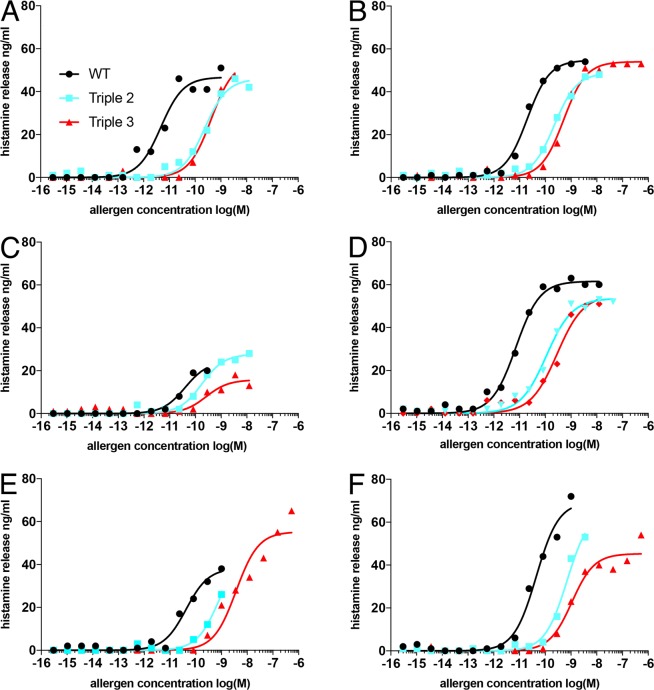
Histamine release (ng/ml, y-axis) induced by rEqu c 1 wt and variants Triple 2 and Triple 3 was measured after passive sensitization of stripped basophils with sera of five horse allergic patients (**A–E**) and RefLab’s positive control serum (**F**) as a function of allergen concentration (x-axis).

The biological activity of rEqu c 1 Triple variants 2 and 3 compared to the rEqu c 1 wild type was analyzed by HRA (Fig. 4). Histamine release was measured after passive sensitization of stripped basophils with sera of six horse allergic persons. Results of HRA show that the biological activity of rEqu c 1 Triple 2 and Triple 3 variants is consistently lower compared to the rEqu c 1 wt with all tested serum samples of horse allergic persons. Calculated *EC*_50_ values (Table 1) were from 5 pM to 45 pM (median 30 pM) for Equ c 1 wt. The values were approximately 8 times higher (110 to 840 pM, median 230 pM) for Equ c 1 Triple 2 variant and approximately 16 times higher (260 pM to 3700 pM, median 470 pM) for Equ c 1 Triple 3 variant suggesting that much higher concentration of Triple 2 and Triple 3 is required for degranulation compared to rEqu c 1 wt. The results show a statistical significant difference between the two variants and wild type Equ c 1 for all sera except for serum C which had the lowest maximum capacity to reduce histamine (Table 1).

## Discussion

### IgE epitope

We obtained very low *IC*_50_ values (21 to 56 pM) for rEqu c 1 wild type in a competitive immunoassay analysis. These values reflect strong binding of Equ c 1 to specific IgE antibodies of serum and is in agreement with previous studies which showed that IgE antibodies exhibit stronger binding to allergens (*K*_*D*_ 10 to 100 pM) compared to IgG binding to antigens (*K*_*D*_ 10 to 100 nM)^6^. Both Triple 2 and Triple 3 variants showed consistently higher *IC*_50_ values compared to wild type, median values being 59 and 191 pM, respectively, except for the serum E which showed similar *IC*_50_ values between Triple 2 and wt. Reduced binding of allergen-specific IgEs to Triple variants 2 and 3 compared to wt allergen suggests that the mutations V47K (in Triple 2) and E21Y (in Triple 3) are located in the IgE epitope area of rEqu c 1 allergen. E21 seems to be more critical for IgE binding because its mutation produced consistently lower binding compared to V47 mutation. Both residues are located on the flat surface of the antiparallel β-sheet of Equ c 1 (Fig. 1B,D). The differences in the results of the competitive immunoassay may reflect heterogenicity of IgE-response among allergic individuals.

This kind of flat or planar shape of an IgE epitope has previously been determined for Bos d 5 and Phl p 2 allergens from the immunocomplex structures between the Fab fragment of an IgE antibody and allergen^6,7^. Because mutations on this surface caused reduction in the IgE binding of sera from five different individuals, it could be argued that this deduced epitope located around V47 and E21 would be common or even generic for an IgE response among horse allergic individuals. Many recent studies have indicated that IgE response utilizes limited germline gene repertoire following somatic hypermutation and affinity maturation^15–17^. It is notable that different individuals seem to utilize similar type of sequences. This implicates that the number of IgE epitopes on the surface of an allergen is more limited compared to the number of IgG epitopes. Especially in the case of small allergens, as Equ c 1, dimer formation provides an optimal orientation of the IgE epitope(s) for recognition and binding by the IgE antibody-FcεRI complex on the effector cell surface^18^. It has been earlier shown by Lascombe *et al*.^14^ that IgE binding of a serum pool of eight horse allergic individuals to Equ c 1 allergen was efficiently inhibited with only one anti-Equ c 1 monoclonal antibody mAb 220 indicating also that number of IgE epitopes of Equ c 1 is restricted.

### Dimerization

One of the benefits of using native MS for studying oligomers is the detection of all oligomeric forms at the same time which is especially beneficial if protein oligomerization is transient and concentration dependent. Size-exclusion chromatography allows the separation of molecules in aqueous non-denaturing conditions based on their molecular size and shape, therefore enabling the separation of protein monomers from their dimers or aggregates without disrupting protein interactions. The native mass spectrometry and SE-UHPLC analyses indicate that rEqu c 1 wt exists predominantly in dimeric form in micromolar and higher concentrations but is able to dissociate to monomers at lower concentrations. These results are in agreement with the results of Gregoire *et al*.^19^ who used ultracentrifugation and gel-filtration chromatography and found the strong tendency for Equ c 1 to form dimer but also reported the dimers can be dissociated to monomers by decreasing the protein concentration or lowering the pH of the buffer solution. The strategy to introduce two alterations on the hydrophobic monomer-monomer interface by replacing two hydrophobic amino acid residues by charged amino acid residues proved to be successful since the dimer/monomer ratio of Equ c 1 was dramatically decreased in Triple 2 and Triple 3 as shown by SE-UHPLC and native mass spectrometric analyses. The very narrow charge state distribution in the native mass spectra shows that Triple 2 and Triple 3 are still very compactly folded. The used monomer mutations would thus be effective in the reduction of cross-linking of specific IgE-allergen complexes on the surface of basophils or mast cells.

### Histamine release assay

*EC*_50_ values calculated from HRA results were extremely low, 5 to 45 pM (median 30 pM) for rEqu c 1 wt indicating that very tiny amount of this major horse allergen can trigger allergic reaction.

*EC*_50_ values has been previously reported in some degranulation studies. Blanc *et al*.^20^ have reported picomolar median values for peanut allergens Ara h 1, Ara h 2, Ara h 3, and Ara h 6, namely 150, 2, 65 and 3 pM, respectively. On the other hand, major birch pollen allergen Bet v 1 seems to trigger degranulation at the higher concentrations, approximately in 2 nM^21^. This may indicate differences in the capacity of natural allergens to cross-link IgEs on the surface of basophils.

The low *EC*_50_ values (5 to 45 pM, median 30 pM) obtained for rEqu c 1 wt is in agreement with low *IC*_50_ values obtained from the competitive inhibition study (21 to 56 pM, median 35 pM), which explains that the potency of rEqu c 1 wt to trigger degranulation at extremely low concentrations is largely based on a strong binding of IgE antibodies to allergen. On the other hand, the increase in *IC*_50_ values was smaller for Triple 2 (1.7 fold increase) and Triple 3 (5.5 fold increase) compared to *EC*_50_ values for Triple 2 (7.7 fold increase) and Triple 3 (15.7 fold increase). Because *IC*_50_ values reflects the binding of IgE antibodies, the higher increase in *EC*_50_ values can be explained by two mutations (V110E + F112K) on the monomer-monomer interface of Equ c 1. Altogether, three point mutations, one on the putative IgE epitope and two on the monomer-monomer interface of Equ c 1 have thus successfully reduced the formation of dimeric allergen-IgE complexes and subsequent cross-linking of IgE on the cell membrane of basophils.

### Recombinant hypoallergen candidates for AIT

As reviewed recently by Valenta *et al*.^1^, recombinant allergen molecules have several advantages over natural extracts in AIT. In addition, it has been demonstrated that full length folded proteins are more effective in developing antibodies that recognize native structures^3^. It can be thus argued that natively folded recombinant allergen molecules or their derivatives with preserved native structure would be promising candidates in AIT. Phase II studies have been reached with recombinant wild type allergens from birch (Bet v 1) and timothy (Phl p 1, Phl p 2, Phl p 5, Phl p 6)^1^. The results have been positive but not so much superior that these products would have been further developed into market.

The key issues in the development of novel recombinant allergens or their derivatives for AIT are their efficacy and safety. One pathway forward to create a safe and efficient hypoallergen is to mutate a limited number of amino acids in allergen affecting its allergenicity yet preserving the genuine folded three-dimensional structure. Thus, the release of allergy mediators from mast cells and basophils and the consecutive appearance of allergic symptoms would be reduced. Instead, protective IgG antibodies against the wild type allergen would be induced. This would prevent mast cell and basophil activation and lead to the modulation of immunoresponse. However, this requires information on critical amino acid (hot spot) residues which especially contribute to the productive formation of allergen-IgE complex on the surface of mast cell or basophil.

Equ c 1 Triple 3 variant that contains three point mutations, one on the putative IgE epitope and two on the monomer-monomer interface, of the major horse allergen Equ c 1 has resulted in reduction of histamine release when using sera from five horse allergic patients. The mutations are not located in the region which was previously reported to include two major T-cell epitopes^12^. Amino acid sequence identity between Equ c 1 Triple 3 and wild type is extremely high, 98.4%, which considerably increases the possibility that IgG antibodies induced by Triple 3 would efficiently bind to Equ c 1 wt. Additionally as shown here, the Triple 3 was recombinantly produced and purified resulting in high purity, homogeneity and correct fold to support the steps toward consistent batch-to-batch manufacturing and safety of Triple 3 as medicinal product. Altogether this would make Triple 3 variant promising candidate for AIT in horse allergy. To evaluate the suitability of Triple 3 further for clinical studies, it would be important to test IgE-binding and histamine release with a wider panel of serum samples of individuals allergic to Equ c 1, to perform T-cell reactivity tests, and continue to animal studies.

## Methods

### Design of variants

Three-dimensional structure of protein molecules were analyzed and visualized using open-source PyMOL 1.7 software. Molecular surfaces were created using UCSF ChimeraX software^22^.

### Cloning, expression and purification of rEqu c 1 allergens

Synthetic gene fragments encoding Equ c 1.0101 (thereafter wild type or wt) and Equ c 1 variants V47K + V110E + F112K (Triple 2) and E21Y + V110E + F112K (Triple 3) with codon optimization for bacterial expression were purchased from GenScript. The synthetic gene fragments of Equ c 1 wt and Triple variants were cloned as NcoI-NotI restriction fragments under the T7 promoter of the pET-28b(+) vector (Novagen) for cytoplasmic expression. The recombinant Equ c 1 allergens contain an extra alanine residue in the N-terminus enabling the usage of NcoI restriction site as well as to keep the open reading frame intact in the cloning of the synthetic genes. The expression vectors were transformed into the *Escherichia coli* BL21(DE3) (Novagen) strain.

Recombinant Equ c 1 wt and Triple variants were produced in 1.8 L (6 × 300 ml) shake flask cultivations in LB medium supplemented with kanamycin (25 µg/ml) and cultivated at +37 °C with 220 rpm shaking until OD_600_ reached ~1. Protein expression was induced by addition of IPTG (isopropyl β-D-1-thiogalactopyranoside) to a final concentration of 1 mM, and cultivation was continued for 4 h at +37 °C with 220 rpm shaking. Cells were harvested by centrifugation (15 min, 5000 rpm. +4 °C). The insoluble protein fraction was isolated from the cell pellet according to Hoffmann-Sommergruber *et al*.^23^ and refolding of the allergen polypeptides according to Arango *et al*.^24^ and Stancombe *et al*.^25^ Briefly, collected bacterial cells were resuspended in 25 mM imidazole, 0.1% TritonX-100 pH 7.4 and lysed by three cycles of freezing and thawing steps. Inclusion bodies were isolated by centrifugation and solubilized in 6 M Urea, 10 mM CAPS pH 10.5 followed by a 3-hour incubation at room temperature under slow stirring. Urea was removed by slowly diluting the solubilization solution 1:10 (v/v) in 10 mM CAPS pH 10.5, after which pH was adjusted to 7.5. Protein was allowed to refold for minimum of 66 hours at +4 °C before concentration with Prep/Scale-TFF 6 ft2 10000 MWCO Cartridge (Millipore).

Recombinant Equ c 1 wt and Triple variants were purified by a standard ion exchange (wild type with HiTrap DEAE followed by a HiTrap CM and variants with a HiTrap Q, GE Healthcare) followed by a size-exclusion chromatography (HiLoad Superdex 75 pg, GE Healthcare). Elution peak fractions after final purification step were analyzed by a Coomassie-stained SDS-PAGE showing that obtained proteins were of high purity and homogeneity. Concentrations of the pure allergens were determined by measuring A_280_ and using sequence-derived extinction coefficient.

### SE-UHPLC analysis

Size-exclusion ultra-high-performance liquid chromatography (SE-UHPLC) analysis of rEqu c 1 wt and Triple variants were performed using Acquity BEH125 SEC column with dimensions of 4.6 × 150 mm, a pore size of 125 Å and a particle size of 1.7 µm (Waters) coupled with Acquity I-Class UPLC instrument (Waters) and controlled by Empower 3 software (Waters). The column was equilibrated to running conditions with PBS (12 mM Na_2_HPO_4_, 3 mM NaH_2_PO_4_, 150 mM NaCl, pH 7.3) as a mobile phase at a flow rate 0.3 ml/min until the baseline was stable. Samples of 2 µl of a 40 µM protein solution were injected. The chromatographic separation was carried out under isocratic flow with overall run time of 12 minutes and detection at 214 nm wavelength. The BEH125 SEC Protein Standard Mix (Waters) was used as a gel filtration standard.

### Mass spectrometry

The mass spectrometric analyses were carried out with a 12 T SolariX XR FT-ICR mass spectrometer, equipped with an Apollo II ion electrospray ionization (ESI) source (Bruker Daltonics, Bremen, Germany). Protein samples were desalted and exchanged into an ESI-compatible ammonium acetate buffer (10 mM, pH 6.9) using PD-10 desalting columns (GE Healthcare). For mass spectrometric characterization in denaturing solution conditions, samples were diluted to the concentration of 0.2 μM with acetonitrile/water/acetic acid solution (49.5:49.5:1.0, v/v). The protein samples were directly infused into the ion source by a syringe pump at a flow rate of 2 μl min^−1^. The temperature and flow rate of the drying gas (N_2_) were 200 °C and 4.0 L/min, respectively, and the pressure of the nebulizer gas was 1.0 bar. Mass spectra were acquired in the positive-ion mode over the m/z range of 800–3000 with 1-Mword time-domain transients and 0.05 s ion accumulation time.

For studying proteins in their native state, the instrumental parameters were carefully optimized to maintain the weak, non-covalent interactions in the gas-phase. Proteins were measured in 10 mM ammonium acetate buffer (pH 6.9). The drying gas temperature was set to 80 °C, and ion accumulation time was 1.0 s. For a native mass spectrum, 100 co-added 262-kword time domain transient were collected and processed to 524-kword data. The wild-type allergen and its Triple variants were measured by using the same instrumental parameters, in order to avoid any bias between the samples. Mass spectra were calibrated externally with respect to the ions of ES Tuning Mix (Agilent Technologies, Santa Clara, CA, USA). Data collection was controlled by ftmsControl software and the data were processed by using Data Analysis software (version 4.4, Bruker Daltonics).

For a determination of the dissociation constant (*K*_*d*_) of the rEqu c 1 wild type allergen, a dilution series with different protein concentrations was prepared. The concentrated stock solution of rEqu c 1 allergen was diluted with 10 mM ammonium acetate to the (monomer) concentrations of 0.1 to 5.0 μM. The slow dissociation of dimers to monomers was taken in consideration by letting the diluted samples to stay four days at +4 °C. The actual concentration of the diluted samples was calculated just before the mass spectrometric analysis by the measured absorbance at 280 nm, using the sequence-derived extinction coefficient. The most diluted samples with concentrations of 0.1 and 0.25 μM showed very low absorbances, suggesting that the protein molecules were partially adsorbed to the walls of the plastic eppendorf tubes. For the less diluted protein samples, five parallel native mass spectra were measured at each different protein concentrations.

The utilization of native ESI-MS for determining monomer-dimer ratio of (transient) protein complexes has been discussed in literature^26^. Briefly, the dissociation constant (*K*_*d*_) was determined from the absolute intensities of ions representing either the monomer (P) or the dimer (P_2_). The total intensities for P and P_2_ were calculated by combining the peak intensities over the charge state distribution (*z* = 1 to *n*) in the ESI-MS spectra. The intensity ratio sufficiently reflects the concentration ratio in solution, if the ionization and transmissions efficiencies are similar for the monomer and dimer. *K*_*d*_ for rEqu c 1 wt dimer was obtained using the procedure described in detail by Niemi *et al*.^9^.

### Competitive IgE binding assay

For the competitive IgE binding assay rEqu c 1 wild type was biotinylated according to the manufacturer’s instructions using a 10-fold molar excess of EZ-Link Sulfo-NHS-LC-Biotin reagent (ThermoFisher Scientific). The biotinylated Equ c 1 wt was spotted as three concentrations (190, 95 and 47.5 µg/ml, 1 nl/spot) onto the streptavidin coated microtiter plate wells (ThermoFisher Scientific) by Nano-Plotter 2.1 (GeSiM). Serum samples from five horse allergic individuals were selected based on their ImmunoCAP ISAC (ThermoFisher Scientific) test profiles. The serum samples used in the competitive immunoassay and histamine release assay were collected from volunteer horse allergic adult (over 18 years) individuals after the permission of the Ethical Committee of Helsinki University Hospital (Finland). The optimal dilution for each serum chosen to be used in the competitive assay was analyzed by IgE serum titration analysis for each spotted concentration (data not shown). For competitive assay, diluted serum was pre-incubated with different amounts of competing allergen in final allergen concentrations of 10 000, 2 000, 400, 80, 16, 3, 0.6 and 0 pM and then added to the well in which the biotinylated rEqu c 1 wt was immobilized. All of the experiments were performed in duplicates.

Peroxidase-conjugated anti-human IgE (Southern Biotech) was used for secondary detection and fluorescently-labelled tyramide (ThermoFisher Scientific) for signal amplification. The fluorescence readout was obtained by scanning the wells with LS400 microarray scanner and 633 nm laser (Tecan). Image analysis, spot detection and fluorescence intensity quantification were performed with Array-Pro Analyzer software (Media Cybernetics). Local background signal value was measured outside the specific spots from each well. The net signal value for each spot was obtained by subtracting the local background signal value from the average intensity value of each spot.

### Histamine release assay

Biological activity of the rEqu c 1 wild type and Triple variants was analyzed by a histamine release assay (HRA). HRA was performed as an outsourced service at RefLab Aps (Copenhagen, Denmark). Briefly, stripped human basophils were passively sensitized with rEqu c 1 wt and Triple variants in 16 different concentrations from 0.05 fM to 0.5 nM (1fg/ml to 10 ng/ml) using the same five serum samples (A-E) as in the competitive IgE binding assay and with an additional positive control serum sample (F) provided by RefLab. Released histamine induced by different allergen concentrations was measured as duplicates by ELISA (enzyme-linked immunosorbent assay) using a glass microfibre method developed by RefLab. In this method glass microfibres coated on microtiter wells bind histamine released from allergen triggered basophils with high affinity and selectivity^27^.

### Statistical analysis

Graphical display, curve fitting, Student’s t-test analysis of competitive ELISA  and histamine release assay data were done with GraphPad Prism v. 8.2.1 (GraphPad Software Inc.). A *P* value of less than 0.05 was considered statistically significant.

### Statement of using human sera

Human participants are not directly involved in this study. The origin of the human serum samples used in the competitive immunoassay and histamine release assay (HRA) and the human basophils utilized in HRA is described as follows. The serum samples used in the competitive immunoassay and histamine release assay were collected from volunteer horse allergic individuals after the permission of the Ethical Committee of Helsinki University Hospital (Finland). Serum samples were collected by Desentum Ltd as an outsourced service using healthcare professionals. The blood samples were numbered and the identity of the donors is known only by the responsible scientist and the healthcare professionals who participated in the collection of the blood samples. Biological activity of the recombinant allergens was analysed by the histamine release assay as an outsourced service at RefLab ApS (Copenhagen, Denmark). RefLab is an accredited laboratory according to: EN ISO/IEC 17025: 2005 and has an in house assay for measuring histamine release from basophils obtained from blood bank buffy coats. Buffy coats were collected at the Blood Bank, National University Hospital of Copenhagen. The Blood Bank has a general ethical approval to hand out buffy coats making sure that the blood donors are anonymous. It is therefore not possible to obtain case history or laboratory case records of the blood donors. RefLab has a contract with the Blood Bank allowing using the buffy coats. The study was conducted in compliance with the Declaration of Helsinki.

## Supplementary information

Supplementary Information.

## References

[1] Valenta R (2018). Allergen extracts for *in vivo* diagnosis and treatment of allergy: is there a future?. J. Allergy Clin. Immunol. Pract..

[2] Akdis CA, Akdis M (2015). Mechanisms of allergen-specific immunotherapy and immune tolerance to allergens. WAO J..

[3] Brown MC (2011). Impact of immunization technology and assay application on antibody performance - a systematic comparative evaluation. PLoS One.

[4] Zieglmayer P (2016). Mechanisms, safety and efficacy of a B cell epitope-based vaccine for immunotherapy of grass pollen allergy. EBioMedicine.

[5] Breiteneder H (2018). Mapping of conformational IgE epitopes of food allergens. Allergy.

[6] Niemi M (2007). Molecular interactions between a recombinant IgE antibody and the β-lactoglobulin allergen. Structure.

[7] Padavattan S (2009). High-affinity IgE recognition of a conformational epitope of the major respiratory allergen Phl p 2 as revealed by X-ray crystallography. J. Immunol..

[8] Rouvinen J (2010). Transient dimers of allergens. PLoS ONE.

[9] Niemi MH (2015). Dimerization of lipocalin allergens. Sci. Rep..

[10] Arsenau AM, Hrabak TM, Waibel KH (2012). Inhalant horse allergens and allergies: a review of the literature. Milit. Med..

[11] Botros HG (2001). Biochemical characterization and surfactant properties of horse allergens. Eur. J. Biochem..

[12] Immonen A (2007). The major horse allergen Equ c 1 contains one immunodominant region of T cell epitopes. Clin. Exp. Allergy.

[13] Gregoire C (1996). cDNA cloning and sequencing reveal the major horse allergen Equ c1 to be a glycoprotein member of the lipocalin superfamily. J. Biol. Chem..

[14] Lascombe M-B (2000). Crystal structure of the allergen Equ c 1. A dimeric lipocalin with restricted IgE-reactive epitopes. J. Biol. Chem..

[15] Kerzel S, Rogosch T, Struecker B, Maier RF, Zemlin M (2010). IgE transcripts in the circulation of allergic children reflect a classical antigen-driven B cell response and not a superantigen-like activation. J. Immunol..

[16] Otte M, Mahler V, Kerpes A, Pabst O, Voehringer D (2016). Persistence of the IgE repertoire in birch pollen allergy. J. Allergy Clin. Immunol..

[17] Croote D, Darmanis S, Nadeau KC, Quake SR (2018). High-affinity allergen-specific human antibodies cloned from single IgE B cell transcriptomes. Science.

[18] Hunt J (2012). A fluorescent biosensor reveals conformational changes in human immunoglobulin E Fc: implications for mechanisms of receptor binding, inhibition, and allergen recognition. J. Biol. Chem..

[19] Gregoire C (1999). Crystallization and preliminary crystallographic analysis of the major horse allergen Equ c 1. Acta Cryst. D.

[20] Blanc F (2009). Capacity of purified peanut allergens to induce degranulation in a functional *in vitro* assay: Ara h 2 and Ara h 6 are the most efficient elicitors. Clin. Exp. Allergy.

[21] Nony E (2015). Development and evaluation of a sublingual tablet based on recombinant Bet v 1 in birch pollen-allergic patients. Allergy.

[22] Goddard TD (2018). UCSF ChimeraX: Meeting modern challenges in visualization and analysis. Protein Sci..

[23] Hoffmann-Sommergruber K (1997). High-level expression and purification of the major birch pollen allergen, Bet v 1. Protein Expr. Purif..

[24] Arango R, Adar R, Rozenblatt S, Sharon N (1992). Expression of *Erythrina corallodendron* lectin in *Escherichia coli*. Eur. J. Biochem..

[25] Stancombe PR (2003). Isolation of the gene and large-scale expression and purification of recombinant *Erythrina cristagalli* lectin. Protein Expr. Purif..

[26] Liu J, Konermann L (2011). Protein-protein binding affinities in solution determined by electrospray mass spectrometry. J. Am. Soc. Mass. Spectrom..

[27] Skov PS, Mosbech H, Norn S, Weeke B (1985). Sensitive glass microfibre-based histamine analysis for allergy testing in washed blood cellsResults compared with conventional leukocyte histamine release assay.. Allergy.

