# Supplementary material for: Development of hypoallergenic variants of the major horse allergen Equ c 1 for immunotherapy by rational structure based engineering
Source: Sci Rep. 2019 Dec 27;9:20148. doi: 10.1038/s41598-019-56812-1 (PMC6934807; doi:10.1038/s41598-019-56812-1)
Supplement: Supplementary file 1 — Supplementary Information. [file 41598_2019_56812_MOESM1_ESM.pdf]

SUPPLEMENTAL INFORMATION

Development of hypoallergenic variants of the major horse allergen Equ c 1 for immunotherapy by rational structure based engineering

Jaana Haka[1], Merja H. Niemi[2], Pekka Mattila[1], Janne Jänis[2], Kristiina Takkinen[3], and Juha Rouvinen[2,*]

1) Desentum Ltd, Kivipylväänkuja 5, 02940 Espoo, Finland
2) Department of Chemistry, University of Eastern Finland, PO Box 111, 80101 Joensuu, Finland
3) VTT Technical Research Centre of Finland, PO Box 1000, 02044 Espoo, Finland

*) Corresponding author
Juha Rouvinen
Phone +358 50 409 6387
E-mail juha.rouvinen@uef.fi

```
                    - -------->                ------>
QQEENSDVAI RNFDISKISG EWYSIFLASD VKEKIEENGS MRVFVDVIRA 50

     ------->      -- ------>      ----->   -------->
LDNSSLYAEY QTKVNGECTE FPMVFDKTEE DGVYSLNYDG YNVFRISEFE 100

   --------->      ---  ----->      =========
NDEHIILYLV NFDKDRPFQL FEFYAREPDV SPEIKEEFVK IVQKRGIVKE 150

           ====
NIIDLTKIDR CFQLRGNGVA QA    172
```

Figure S1: Amino acid sequence of the Equ c 1 allergen. The secondary structure elements β-strands (—>) and α-helices (===) are indicated above the sequence. The published T-cell epitope region is underlined. Amino acid residues targeted to mutagenesis are bolded and colored, IgE-epitope residues in blue and monomer residues in red.

A

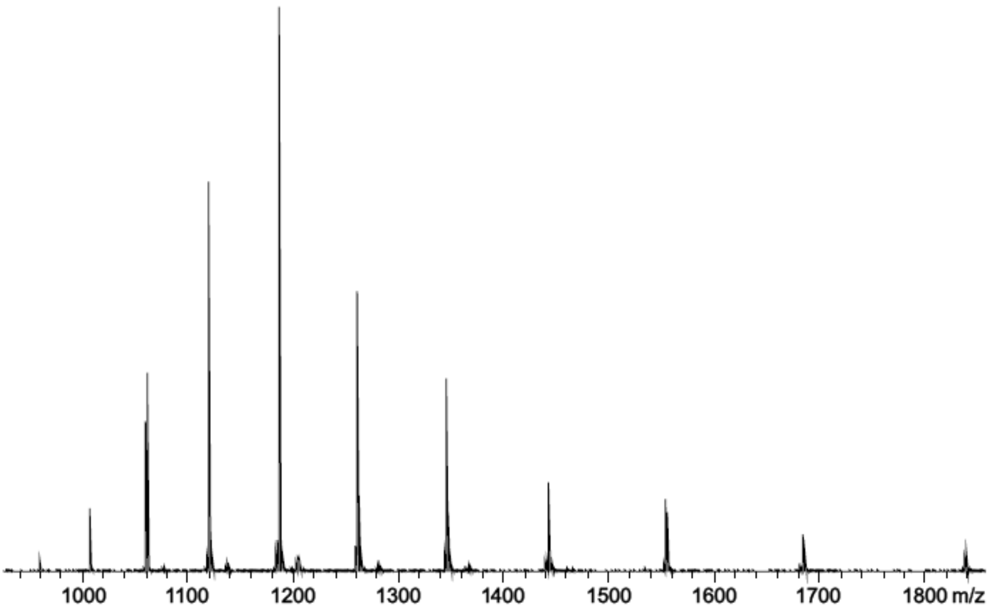

B

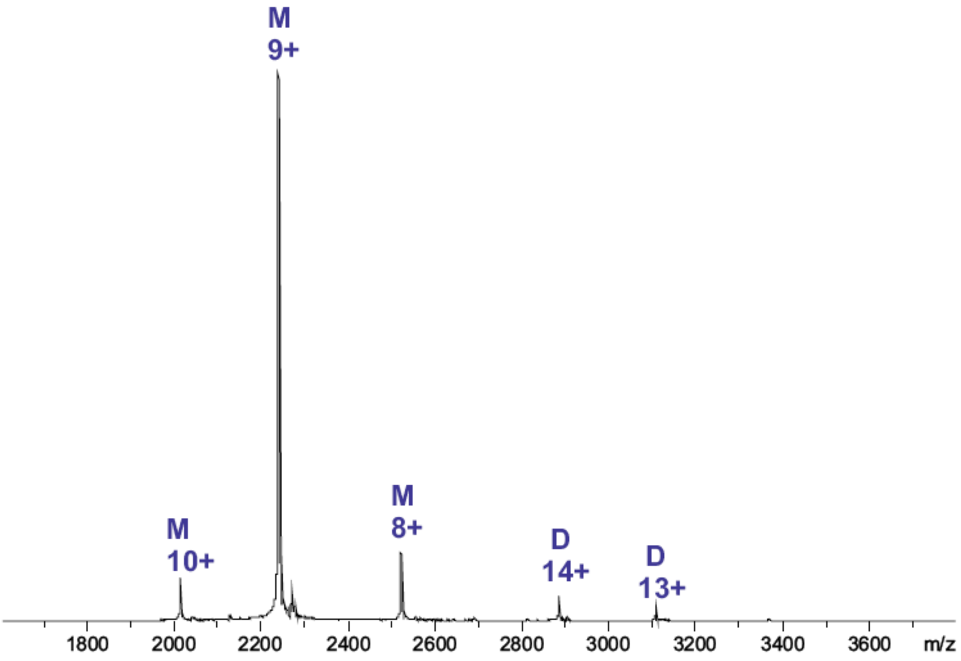

Figure S2: The high resolution mass spectra for Triple 2 in denatured (A) and native form (B). Numbers refer to the charge states. Native spectra were measured at 40 µM protein concentrations and monomeric and dimeric peaks are labelled (M or D).

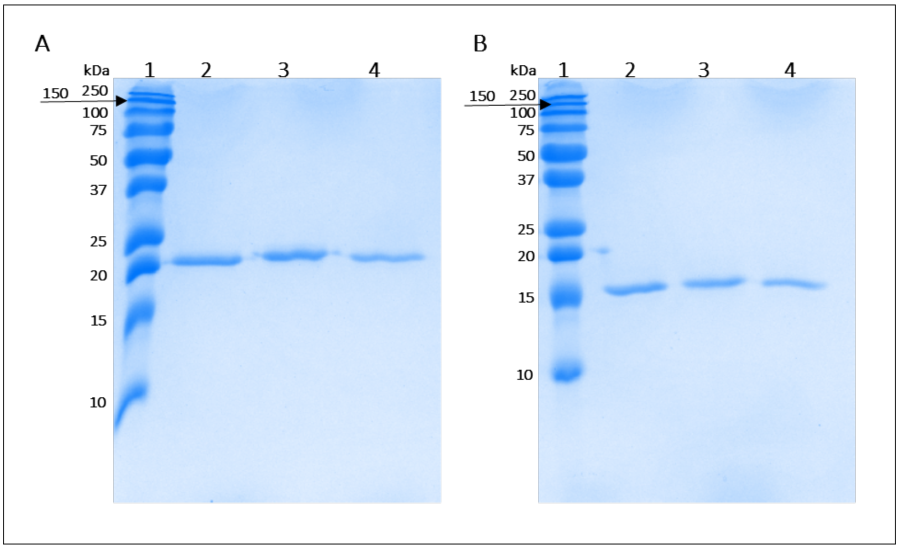

Figure S3: Coomassie stained 15% SDS-PAGE analysis of the purified Equ c 1 wt, Triple 2 and 3 in (A) reducing conditions and (B) non-reducing conditions. Samples containing 1.5 µg of Equ c 1 wt, triple 2 and Triple 3 were loaded in the slots. Lane 1: Molecular weight marker: Precision Plus Protein Dual Color Standards (BioRad), Lane 2: Equ c 1 wt, Lane 3: Triple 2 and Lane 4: Triple 3. The pictures of the Coomassie stained gels were taken with a GelDoc™ XR+ Imaging System (BioRad).

In the reducing conditions Equ c 1 allergens (wt, Triple 2 and 3) migrate with a molecular weight of ~ 22 kDa (Fig. S3 A). In the non-reducing conditions Equ c 1 allergens migrate with a molecular weight of ~15 kDa (Fig. S3 B). In the non-reducing conditions the disulphide bridge of Equ c 1 allergens is intact and thus folding is more compact resulting in faster migration in the 15% SDS-PAGE compared to reduced Equ c 1 allergens. In the non-reduced Equ c 1 allergens dimeric forms are not observed due to SDS in the polyacrylamide gel.

Similar difference in the migration rate in the SDS-PAGE analysis of reduced and non-reduced of Equ c 1 wt allergen has earlier been observed also by Botros et al. 2001.[11]
